# Un-Meetings as tools for translational idea generation: A semantic content analysis of an Opioid Crisis Un-Meeting

**DOI:** 10.1017/cts.2022.490

**Published:** 2022-11-09

**Authors:** Reza Yousefi Nooraie, Robert J. White, Scott Steele, Erika F. Augustine, Deborah J. Ossip, Martin S. Zand

**Affiliations:** 1 Department of Public Health Sciences, University of Rochester Medical Center, Rochester, NY, USA; 2 Center for Leading Innovation and Collaboration (CLIC), Clinical and Translational Science Program National Coordinating Center, University of Rochester Medical Center, Rochester, NY, USA; 3 Kennedy Krieger Institute, Baltimore, MD, USA; 4 Division of Nephrology, Department of Medicine, University of Rochester Medical Center, Rochester, NY, USA

**Keywords:** Un-meeting, team science, content analysis, opioid crisis, network analysis

## Abstract

**Background:**

Team development and idea generation are key intertwined steps in translational science that need a framework to accommodate unstructured, participatory interactions. To this end, we introduced Un-Meetings to the Clinical and Translational Science Awards (CTSA) Program, innovative events that facilitate cross-disciplinary idea generation and informal discussions between translational scientists, policy makers, community members, advocates, and public health professionals. Here we describe a mixed methods study to characterize the conceptual diversity and clusterization of ideas generated through an Opioid Crisis Un-Meeting.

**Methods:**

An Un-Meeting targeting translation science approaches to the opioid crisis were hosted at the University of Rochester Center for Leading Innovation and Collaboration (CLIC). We used semantic analysis and conceptual mapping of keywords to analyze how attendee-led idea generation sessions identified topics for breakout discussions.

**Results:**

One hundred and two individuals from 40 institutions proposed 150 unique ideas that were grouped into 23 breakout sessions. Network analysis showed that diverse pools of experts were bridged by topics addressing the complexities of the opioid crisis. Two clusters emerged: (1) systems, contexts, and community engagement, and (2) technologies, innovations, and treatment advancements.

**Conclusions:**

The cross-disciplinary nature of topic areas that bridge across thematic communities provide opportunities for CTSA programs to engage and support development of diverse translational teams. Potential opportunities for team building include technological advancements of opioid prevention, treatment, surveillance, systems approaches, and studies focusing on special populations and health disparities. The analysis method here may be useful in identifying naturally emerging teams of experts and community gaps when addressing large problems.

## Introduction

Translational research involves bridging between different stages of the translational spectrum (e.g. laboratory, clinic, community) to find, test, and implement solutions that address complex biomedical and health problems [1]. Innovative ideas are created by teams of multi-disciplinary researchers and stakeholders. There are only a few reports on the impact of scientific retreats [2,3], conferences, and other participatory activities [4] designed to motivate and facilitate idea generation and team building. Despite conceptual advances in understanding determinants of successful translational collaboration [5], little has been done to intentionally create environments that facilitate development of translational ideas and creation of translational research teams, especially at the level of large research consortia.

The *Un-conference* is an innovative meeting that shifts from traditional didactic lectures and presentations to a more participatory format that engages attendees from multiple disciplines to co-develop ideas and form collaboration networks [6]. Un-conferences facilitate cross-disciplinary idea generation, informal conversation, and formation of professional collaboration networks. Un-conferences differ from frameworks designed to arrive at group consensus among experts (e.g. Delphi Method [7]), which are designed to arrive at a consensus about an issue, rather than form teams and collaborations to address an issue using novel approaches. The agendas for sessions and new collaborations emerge from unstructured, organic, interest-based, group discussions. This format has been highly successful within the technology sector [8,9].

The University of Rochester Center for Leading Innovation and Collaboration (CLIC), the National coordinating center for the National Institutes of Health Clinical and Translational Science Awards (CTSA) Program, adapted the methodology of Un-conferences [6] to develop Un-Meetings, which are designed to intentionally foster translational innovation, idea generation, and spark team collaborations, as previously been reported in detail [10]. Several Un-Meetings have been hosted by CLIC during last few years, which provided opportunities for idea generation and free conversations by multi-disciplinary panels of investigators and research users, on different translational topics, including healthcare workforce development [10], rural health and equity, artificial intelligence, life course research, academic-community hospital partnership development, and climate change [11]. Here we use the Un-Meeting framework [12], a vehicle to support cross-disciplinary team formation and innovation, and a semantic network analysis method for studying these processes in the clinical and translational science community.

The unstructured format of the Un-Meeting allows researchers to form social clusters that may lead to early transdisciplinary team formation. In addition to formal organizational affiliations (e.g. institutions and departments), invisible communities of research and collaboration can form based on shared interests, values, and scientific worldviews that cross traditional organizational structures. These “invisible colleges” [13], “invisible communities” [14], or “epistemic communities” [15] are difficult to identify and often are only nascent or yet-to-be-formed communities waiting for a catalytic event. However, they are critical in knowledge creation and formation of new disciplines [16]. Thus, identifying invisible research communities is critical for translational research and for addressing inherently multi-factorial and complex research problems (e.g. obesity [17], addiction [18], or implementation science [19]) that span across biological, preclinical, clinical, public health, and socio-cultural realms.

The data collected through an Un-Meeting provide a unique opportunity to study the emergence of translational ideas for future research from the perspective of investigators interested in developing emerging collaborations, and to identify potential invisible research communities. Previous efforts identifying such ideas and communities have been largely limited to semantic analysis of translational research topics, heavily focused on bibliometric analysis of research outputs to identify hot topics and emerging fields (e.g. cancer research, heart disease [20]) or common research approaches (e.g. epidemiology, pharmacokinetics [21]). By focusing on the products of collaborations, such efforts miss the opportunity to identify cross-disciplinary potential teams, collaborations, and factors influencing team formation.

In contrast, analytic clustering of dynamic Un-Meeting topic keyword data allows a multifaceted analysis of attendee interests, dominant fields, and common frameworks. Such keyword analysis may identify bridging topics and cross-disciplinary approaches for future scientific team formation to address urgent research problems. Network analysis can identify topics that connect attendees from disparate research areas, similar to identifying bridging keywords in research publications that cross scientific fields [22–24].

In this exploratory study, we describe content analysis of topics generated by participants in an Un-Meeting on the “opioid crisis” at University of Rochester. We investigated the clustering patterns and bridging action words, as indicators of dominant fields and bridging opportunities.

## Methods

### Un-Meeting Planning and Data Collection

The Center for Leading Innovation and Collaboration (CLIC), housed at the University of Rochester, hosted the first CTSA-wide “Un-Meeting” on June 2, 2018 addressing the opioid crisis. The Un-Meeting is a participatory event focused on engaging translational scientists, policy makers, community members, advocates, and public health professionals in a dynamic process of idea generation and translational conversation [25]. The Un-Meeting began with an introduction to the objectives and event rules, followed by a series of lightning talks (“4×4s”; 4 minutes, 4 slides) by scientists and decision-makers, aiming to provide a primer for potential topics related to the opioid crisis for subsequent small group breakout sessions [12].

After the talks, attendees used sticky notes to write topics of interest, questions, or ideas for breakout sessions and placed them on an idea generation board. Assisted by the organizing team, conference participants were encouraged to group the ideas into common themes that would determine the eventual topics for the subsequent concurrent small group breakout sessions. Facilitators and participants re-arrange and aggregate these notes into small group session topics in specific rooms and time slots. Attendees then self-select which sessions they would like to join. The resulting breakout sessions were 45 minutes long, unmoderated, and included between 8 and 25 participants. A second round of 4×4 talks, topic generation, and breakout sessions occurred in an afternoon session.

### Topic Mapping

Photographs of the notes posted on the grid were used to collect the final clustering, and were transcribed into machine-readable format by hand. Each note was treated as a separate unit of text and was pre-processed by removing stop words (e.g. the, and, has) and common terms using the Automap program [26]. The resulting keyword list was manually examined, and obvious synonyms were merged.

We used two analytical techniques to explore the patterns of topic clusterization:

*Keyword cluster analysis:* We clustered keywords based on their co-occurrence in notes and linked them with their corresponding breakout sessions using a density-based spatial clustering (DBSCAN) algorithm [27] with a centroid cluster dissimilarity function (Mathematica version 10.1; Wolfram, Indianapolis IN). The algorithm grouped together keywords on a two-dimensional surface based on commonality of their neighbors. The resulting clusters had keywords that were used together and shared similar neighboring keywords. In order to visualize the extent to which these clusters resemble the resulting breakout sessions, we developed a heatmap showing the commonality of keywords in clusters and breakout sessions.

*Keyword-to-session network mapping:* To identify keywords that bridged across fields and disciplines, we performed a separate analysis on keywords that were assigned to more than one breakout session. We expected that this analysis would provide clues to translational opportunities to address the opioid crisis and opportunities for cross-disciplinary team formation. We developed a keyword-to-session two-mode matrix and map, with nodes representing keywords and breakout sessions, and edges representing the relation between keywords and sessions. The nodes (keywords and sessions) were arranged in a two-dimensional graph using multi-dimensional scaling (MDS) of geodesic distances between nodes [28] and plotted using NetDraw [29].

## Results

### Participant Characteristics

In total, 102 individuals from 40 institutions (47% academic, 27% CTSA hub affiliate, 3% NIH, 11% other government agencies, 5% community organizations, 1% foundation, and 6% other) participated in the Un-Meeting. A third of participants came from within a 100-mile radius of the Un-Meeting location, the remainder represented 16 states and the District of Columbia.

### Keyword Cluster Analysis

The goal of this analysis was to visualize the conceptual distance between and within breakout sessions. Such a mapping can show the conceptual diversity of the conference, grouping of words within the breakout sessions, provide an unsupervised clustering window into the breadth or focus of a particular breakout, and cross-connections between breakouts.

**Fig. 1.**
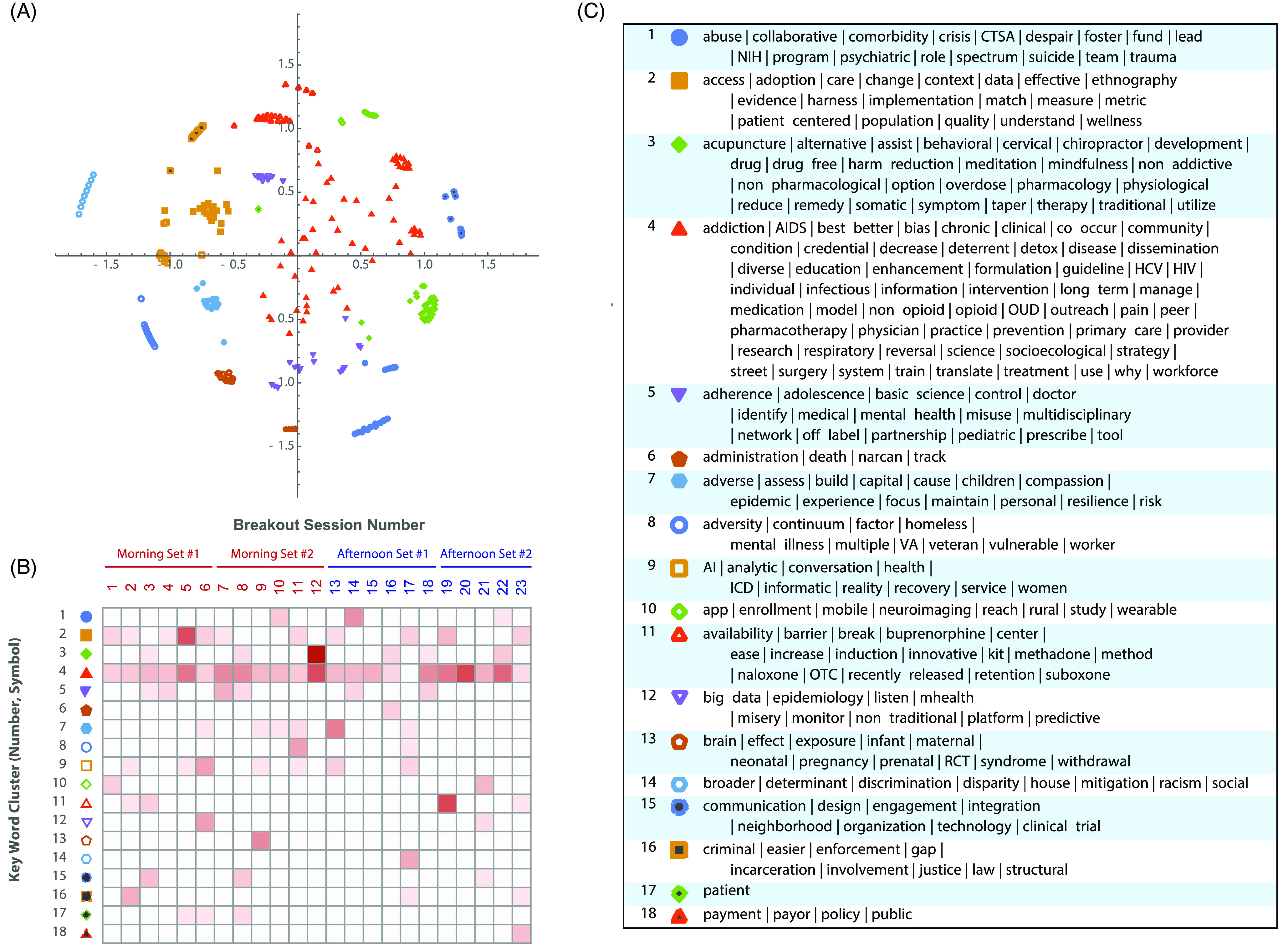
Keywords cluster analysis. (A): the clustering map; (B): keyword cluster to breakout session heat map; (C): the list of keywords in each cluster.

As a result of the idea generation session, 150 unique ideas were documented and grouped into 23 breakout sessions (Supplementary table 1). Unsupervised clustering of the 248 keywords in 150 sticky note ideas resulted in 18 clusters (Fig. 1A). The heatmap in Fig. 1B represents how frequently keywords within each cluster overlapped between breakout sessions. For example, the keywords in Cluster 4 were widely distributed across almost all sessions (Fig. 1B). This likely reflects the general medical theme of the keywords in this cluster, including ‘opioid’ (n = 39 occurrences), ‘treatment’ (n = 27), ‘pain’ (n = 15), ‘research’ (n = 8), ‘medication’ (n = 8), and ‘community’ (n = 8). Many less frequent, but conceptually distinct, keywords were also included in this cluster. Examples include ‘dissemination’ (n = 1), ‘outreach’ (n = 1), ‘workforce’ (n = 1), and ‘translate’ (n = 2). Similarly, cluster #2 keywords occurred broadly across 11 sessions, and included topics related to adoption/implementation/quality and evaluation: ‘access’ (n = 12), ‘implementation’ (n = 4), ‘context’ (n = 3), and ‘quality’ (n = 2). On the other hand, clusters with unique or limited multi-session occurrences included keywords such as ‘neonatal’ (n = 1), ‘prenatal’ (n = 1), ‘discrimination’ (n = 1), ‘disparity’ (n = 1), ‘racism’ (n = 1), ‘payment’ (n = 1), and ‘policy’ (n = 2).

We next examined the linkage between breakout sessions and keyword clusters. Breakout sessions also varied in terms of the breadth of the distribution of their keywords across clusters. Six sessions with highest between-cluster breadth shared keywords with five or six clusters. They included *Disparities in Addiction*, Access to Treatment (Session 17), *Criminal Justice* (Session 2), *Clinical trials* (Session 3), *Community Engagement* (Session 8), *Special Populations* (Session 11), and *Health Insurance/Policy* (Session 23). Most sessions addressed systemic and cross-disciplinary issues, involving community, governmental, and policy sectors.

Most interestingly, we found that the content of breakout sessions rarely corresponded with keyword clusters, as the content of clusters usually referred to multiple breakout sessions. Thus, clusters related to general medical aspects of addiction and systemic and organizational themes distributed across breakout sessions. Themes related to special populations and disparities did not appear in many sessions and were more specific to the sessions corresponding to these topics.

### Keyword-to-Session Network Mapping

**Fig. 2.**
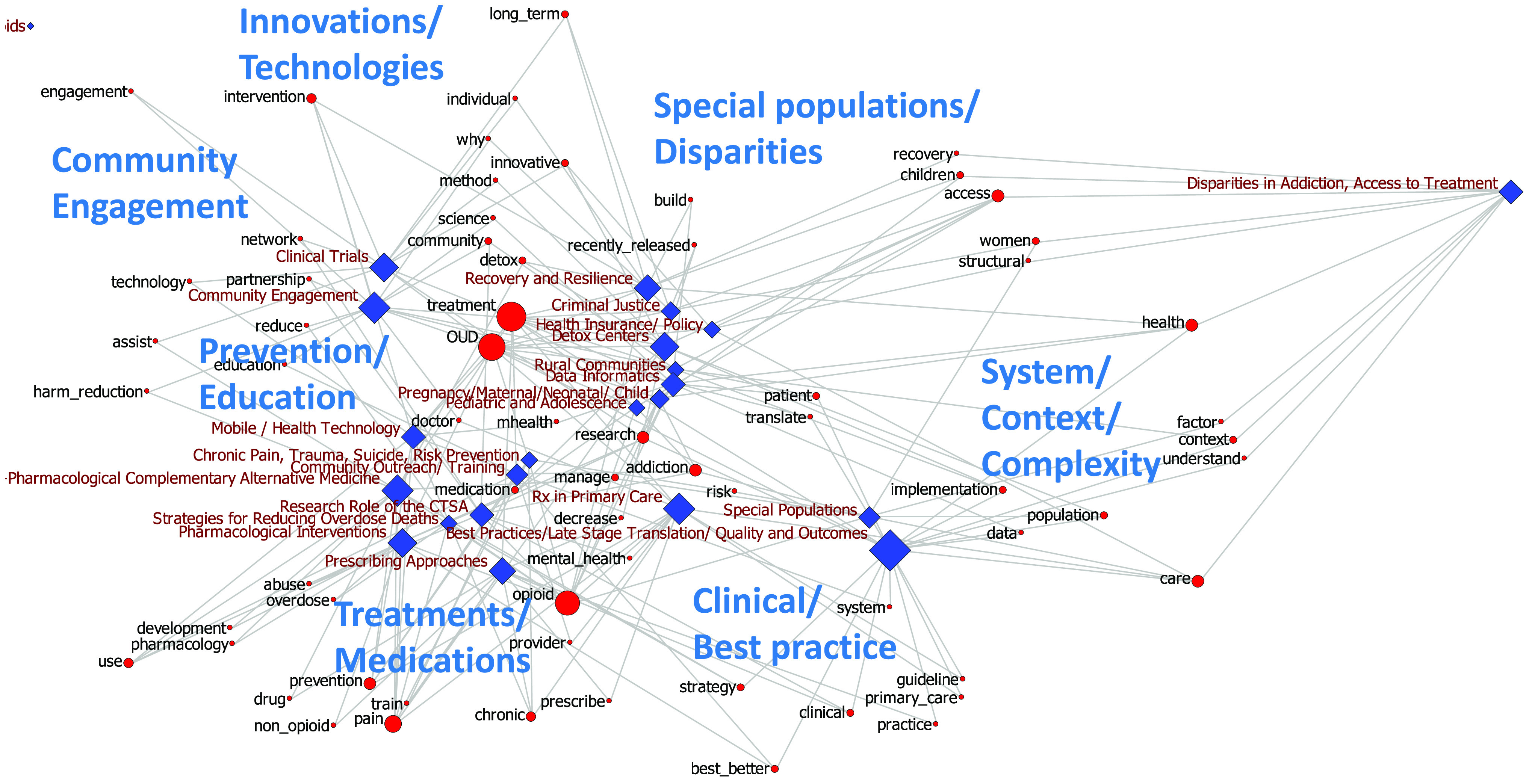
The shared keyword-to-session map with MDS algorithm. Blue nodes: breakout sessions; red nodes: keywords; blue text: thematic regions; node size corresponds to the number of connections.

We next analyzed the relationship between keywords and sessions, based on their distribution in a network map, aiming to identify meaningful regions and distinct communities. Figure 2 shows the distribution of keywords (red circles) that were shared between sessions (blue diamonds) in the keyword-to-session network. As expected, given the MDS layout method, keywords shared by broader sessions concentrated around the center of the network map (e.g. “treatment,” “OUD,” “doctor”), while others that prominently bridged across various regions of the map appeared at the periphery.

After reviewing, the common features of keyword clustered in different regions of the map and sessions that they bridged. We labeled seven main regions, denoted by the large blue labels in Fig. 2. The labeling process was a subjective activity to find labels that meaningfully describe the main themes of keywords and sessions in each region, and to identify thematic regions of the map. These regions were labeled as **‘community engagement’** (including keywords such as ‘engagement’, ‘partnership’, ‘network’), **‘technologies and innovations’** (including keywords such as ‘intervention’, ‘innovative’, ‘science’, ‘why’, and ‘methods’), **‘treatments and medications’** (including: ‘drug’, ‘pharmacology’, ‘development’, ‘prescribe’, ‘overdose’, ‘abuse’, ‘pain’), **‘clinical and best practice’** (including ‘guideline’, ‘clinical’, ‘best/better’, ‘practice’, ‘primary care’), **‘prevention and education’** (including ‘education’, ‘harm reduction’, ‘assist’), **‘special populations and disparities’** (including ‘recently-released’, ‘children’, ‘women’), and **‘system/context/complexity’** (including ‘structural’, ‘implementation’, ‘context’, ‘translate’, ‘population’, ‘data’).

In summary, we identified thematic regions in the map that can indicate to potential/invisible communities among researchers with common interests and approaches in opioid research. The main clusters included clinical research, translational research using technologies, population health, community engagement, special populations and disparities, and systemic and structural approaches to opioid disorders.

## Discussion

In this report, we describe the use of unsupervised clustering based on semantic analysis of concepts and keywords to identify bridging concepts across expert domains and hidden communities of interest within an Un-Meeting involving investigators and research users interested addressing the opioid crisis. We used two complementary approaches to identify clusters of keywords and illustrated the overlap between sessions and keyword clusters, and areas that bridged across potential communities of research. We found that keywords related to treatment, pain, medication, access, and communities span across many breakout sessions that represented focused areas of investigation, while important concepts such as disparity, discrimination/racism, and policy were less frequent and were limited to few focused communities. The network analysis showed that focused communities were bridged by topics pertaining to the complexities of the opioid crisis, including systems, contexts, and community engagement, on one hand, and technologies, innovations, and treatment advancements on the other hand. These bridging concepts represent translational opportunities in opioid research that connect disciplines and research groups.

Results of the thematic analysis confirmed the conceptual diversity, with a majority of breakout sessions related to more than one topic area/disciplinary field, and spanning socio-political, technological, and clinical disciplines. This supports the translational nature of opioid research, similar to many other complex fields, in which, the expertise and research focus could not be easily classified by traditional departmental and disciplinary boundaries [30]. This underscores the importance of identification and support of invisible/epistemic communities focusing on different aspects of this research, and development of strategies to bridge across them to facilitate knowledge translation and cross-disciplinary collaboration.

The opioid crisis affects several aspects of healthcare systems, spanning from prevention and public health to emergency care, and from primary care to specialties. The opioid crisis is even more complicated by a gradual change in the demographics of those most affected, and emergence of new behavioral trends [31,32]. These complexities underscore the need for transdisciplinary collaboration between researchers and knowledge users (e.g. policy makers, community-based organizations, healthcare providers) at different stages of translational spectrum [33,34], providing a much-needed and fertile opportunity for CTSA researchers and their institutions to promote translational scientific collaborations and innovations [35]. Major bridging topics identified in our analysis could be mapped to areas of current CTSA Program emphasis (e.g. community engagement, methods and processes, informatics, and clinical trials) [36] and the emerging area of dissemination and implementation [37] and health equity [38]. The bridging regions of the keyword map in our analysis are also consistent with the major areas of capacity building by CTSA hubs to address the opioid crisis, identified by Cottler et al. who classified them into *preclinical*, *clinical research, community engagement, computational science and data informatics*, and *workforce development and implementation science* [35]. While it is likely that this reflects the fact that over 75% of the conference attendees came from the CTSA consortium, this also provides an opportunity to identify potential areas for scientific team formation.

Inspection of the bridging concepts in the keyword-to-session map provides clues for translational opportunities in opioid research to be fostered by CTSA programs. These opportunities include:

- Collaboration between clinical and basic sciences researchers to develop new medications and treatments. This is a dominant area in the center of Fig. 2, that involves themes related to innovative treatments, use of technologies to develop innovative methods of research and evaluation, and data science.
- Collaboration between health researchers and community members as well as community-based institutions, to engage stakeholders in research and to address disparities. The **Community engagement** region in Fig. 2 connects themes related to treatment, prevention, education.
- Collaboration between clinical researchers, system scientists, dissemination & implementation scientists, and sociologists to study the complexities of social systems and organization of care. The region labeled **System/Context/Complexity** in Fig. 2 is located adjacent to **Special populations/Disparities**, which underscores the role of systemic approaches to improve health equity in this area.

While the results of our study at a minimum provide a potential roadmap of opportunities for translational advancement in opioid research among this core subgroup of scientists, there are several limitations to be acknowledged. The keywords used in the mapping are based on limited content provided on sticky notes and might miss the complexities and breadth of subject matters to which they correspond. This may affect the richness of conceptual clusters and resulting interpretations. The assignment of topics to breakout sessions was moderated by a facilitator and hence does not reflect a completely unsupervised process of clustering. The topics also reflect the areas of expertise of this particular group of attendees and may not be generalizable. Nevertheless, this method of semantic analysis with thematic network analysis and keyword clustering may be useful for analyzing Un-Meetings with the aim of identifying hidden communities, areas of potential collaboration, and potential gaps in a research network’s ability to impact a chosen translational research problem. The value of these clustering techniques in identifying emerging communities and future collaboration potentials could be assessed using mixed methods studies to incorporate the perspectives and reflections of investigators involved in idea generation activities into the analysis.

## Conclusions

Our case study of mapping the generated ideas in an Un-Meeting on the opioid crisis, an inherently translational issue, provided insights on the thematic clusters and topic areas that bridge across them. Our findings add to the scientific body of literature on using thematic mapping to reveal the complexities of inter-disciplinary research [39] and provide clues about emergent themes to inform strategic planning in translational research [40]. The dynamic process of grouping breakout session ideas into clusters is similar to other graphical approaches used to classify concepts and topics, such as Concept Mapping [40–42]. The Un-Meeting format appears particularly suitable to study the dynamics of idea generation and team building in translational science, where transdisciplinary teams are critical to advance novel discoveries and innovative solutions to biomedical problems across multiple “translational barriers” via cross-disciplinary teams [5,43].

The cross-disciplinary nature of topic areas that bridge across thematic communities provide opportunities for CTSA programs to engage and support diverse communities of researchers and users to develop translational teams. Potential opportunities to support translational collaborations include technological advancements of opioid prevention, treatment, and surveillance; studying systems, contexts, and complexities of the opioid crisis, and studies focusing on special populations and health disparities.

## Supplementary material

For supplementary material accompanying this paper visit https://doi.org/10.1017/cts.2022.490. click here to view supplementary material
