# Supplementary material for: Un-Meetings as tools for translational idea generation: A semantic content analysis of an Opioid Crisis Un-Meeting
Source: J Clin Transl Sci. 2022 Nov 9;6(1):e124. doi: 10.1017/cts.2022.490 (PMC9794961; doi:10.1017/cts.2022.490)
Supplement: Supplementary file 1 [file S2059866122004903sup001.docx]

**Appendix 1 - Idea Generation & Breakout Sessions**

| **Session** | **Ideas Generated (verbatim text)** | **Breakout Room Topic** |
| --- | --- | --- |
| 9:00 am – 10:45 am | - Access / reaching rural communities - OUD in rural contexts - Rural OUD treatment implementation | S1: Rural Communities |
| 9:00 am – 10:45 am | - SUD in recently released women from incarceration - Why the treatment gap? - Easier access for long term treatment - Criminal Justice involvement - Law enforcement | S2: Criminal Justice |
| 9:00 am – 10:45 am | - Innovative methods to engage individuals with OUD in research - Clinical Trials networks - Technology assisted interventions for long term treatment of OUD - Innovative Clinical Trials - Innovative Trial Design | S3: Clinical Trials |
| 9:00 am – 10:45 am | - Pediatric Addiction - Opioid addiction in adolescence - Pediatric - Medication treatment for OUD access adherence | S4: Pediatric and Adolescence |
| 9:00 am – 10:45 am | - Implementation of effective treatments for OUD, matching patients to treatment - Health care systems change - Application of implementation research to harness data and translate best evidence into practice - Patient-centered treatment + strategies - Quality measures, for treatment of OUD - Quality of care metrics - Primary care / clinical guideline adoption - “Ethnography:” understanding populations and context | S5: Best Practices/Late Stage Translation/ Quality and Outcomes |
| 9:00 am – 10:45 am | - Following patients (through treatment) - Non-traditional data sources - ICD codes and reality - Predictive analytics - Build an AI-powered listening platform to monitor conversations about opioids - Data Analytics for Epidemiology - Misery - Predictive analytics / AI - Health services research - Big data - Data science / Informatics / Health | S6: Data Informatics |
| 10:45 – 11:30 am | - Tools for providers to identify OUD - Primary Care - Multidisciplinary approach to treatment of chronic pain - Treating OUD in Primary Care - Opioid prescribing guidelines for doctors - Medical and Mental Health Care - Prevention of misuse/SUDs | S7: Rx in Primary Care |
| 10:45 – 11:30 am | - Community-based approaches - Integration and translation of OUD treatment to communities - Educating patients about why this is important - Science Communication: community, patients, doctors - Partnering with existing community organizations - Harm reduction and neighborhoods - Education - Community engagement - Community-based interventions | S8: Community Engagement |
| 10:45 – 11:30 am | - Opioid effects on Infant’s brain, research - Pregnancy - Addiction in pregnancy - NAS management, RCT - Opioid use for infants and children - NOWS, Neonatal Opioid Withdrawal Syndrome - Prenatal exposure - OUD and maternal health | S9: Pregnancy/Maternal/Neonatal/ Child |
| 10:45 – 11:30 am | - Trauma opioid use - Despair - Psychiatric comorbidity and OUD - Opioid use d/o + - Chronic Pain - Suicide risk/prevention | S10: Chronic Pain, Trauma, Suicide, Risk Prevention |
| 10:45 – 11:30 am | - Opioid and vulnerable populations (including workers) - Opioid use in women - VA’s continuum of care to manage Veterans opioid addiction - High risk populations, homeless, mental illness - Multiple risk factors/adversities | S11: Special Populations |
| 10:45 – 11:30 am | - Non-Pharmacological approaches to Pain - Non-Pharm treatments - Alternative treatments (drug-free) to manage pain - Behavioral therapy for pain management in place of drug therapy - Acupuncture “detox,” harm-reduction” - Mindfulness and meditation for pain management - Advancing the development of non-addictive pain remedies - Somatic/physiological treatment - Strategies utilizing alternative therapies in assisting opioid taper - Pain prevention symptom management - Treatment options including meds and non-pharm options - Use of upper cervical chiropractors to address pain vs. traditional chiropractors | S12: Non-Pharmacological Complementary Alternative Medicine |
| 1:00 – 1:45 pm | - Recovery capital, assessing, building - Long-term interventions what to do after detox - Resilience - Maintaining personal wellness for those tackling the epidemic - Health / wellness of individuals in recovery - Recovery - Opioid use disorder and other substance use - Less focus on cause of addition, more focus on treatment recovery - Compassion - ACE’s, Adverse Childhood Experiences | S13: Recovery and Resilience |
| 1:00 – 1:45 pm | - Proper funding for mental health and substance abuse/addiction - Approaches to foster new collaborative clinical/research teams and networks - Community and research partnerships - Role of CTSA program + NIH in leading the full spectrum of research in opioid crisis | S14: Research Role of the CTSA |
| 1:00 – 1:45 pm | - Infectious diseases - HCV/HIV - HIV/AIDS as co-occurring condition | S15: Infectious Diseases and Opioids |
| 1:00 – 1:45 pm | - Pain and overdose - Strategies for reducing deaths from Opioid overdoses - Narcan administration and tracking | S16: Strategies for Reducing Overdose Deaths |
| 1:00 – 1:45 pm | - Recovery housing for women with children - Understanding disparities and access to care - Social-structural factors/broader contexts - Racism discrimination - SDOH mitigation | S17: Disparities in Addiction, Access to Treatment |
| 1:00 – 1:45 pm | - Chronic pain and Opioids - Opioids after surgery - Non-opioid pain treatment, off-label drugs, basic science - Prescribing practices and training approaches - Decreasing opioid prescription in clinical settings- it is not well controlled | S18: Prescribing Approaches |
| 2:00 – 2:45 pm | - Increase Availability to Detox - Decrease Barriers to Detox - Increase/Ease access & number of Detox Centers - Innovative methods for MAT induction – in the field - MAT in recently released populations - How do we break down barriers to MAT implementation? - Access to naloxone kits (OTC) - Buprenorphine for pain +/- OUD - Increase access to suboxone - Increase access to medication assisted treatment - Methadone treatment access and retention | S19: Detox Centers |
| 2:00 – 2:45 pm | - Street Outreach - Prevention - Information Dissemination - Addressing biases in physicians through better education - Use of peers - Socioecological model - Educating and training a diverse workforce to address addiction - Appropriately credentialed providers in treating addiction | S20: Community Outreach / Training |
| 2:00 – 2:45 pm | - Wearables / mHealth - Use of mobile technology to treat/prevent OUD - Mobile approach to study enrollment - mHealth systems for chronic pain - app based interventions - neuroimaging | S21: Mobile / Health Technology |
| 2:00 – 2:45 pm | - Pharmacology formulation & research - Medication development for OUD - Medication development for overdose reversal - Non-opioid pharmacotherapy for pain - How to best use abuse deterrent formulations of opioids - Medication development respiratory enhancement | S22: Pharmacological Interventions |
| 2:00 – 2:45 pm | - Public policy - Innovative payment structures - Payors are making the problem worse - Policy and access - Access to care - Payors and OUD treatment access | S23: Health Insurance / Policy |
